# Coupling between Land–Ocean–Atmosphere and Pronounced Changes in Atmospheric/Meteorological Parameters Associated with the Hudhud Cyclone of October 2014

**DOI:** 10.3390/ijerph15122759

**Published:** 2018-12-06

**Authors:** Akshansha Chauhan, Rajesh Kumar, Ramesh P. Singh

**Affiliations:** 1 Department of Environmental Science, School of Basic Sciences and Research, Sharda University, Knowledge Park-3, Greater Noida 201306, India; akshansha1886@gmail.com (A.C.); rajeshkrsh.in@gmail.com (R.K.); 2 School of Life and Environmental Sciences, Schmid College of Science and Technology, Chapman University, One University Drive, Orange, CA 92866, USA

**Keywords:** Hudhud cyclone, AIRS, atmospheric and meteorological, ozone, cyclone

## Abstract

India is vulnerable to all kinds of natural hazards associated with land, ocean, biosphere, atmosphere, and snow/glaciers. These natural hazards impact large areas and the population living in the affected regions. India is surrounded by ocean on three sides and is vulnerable to cyclonic activities. Every year cyclones hit the east and west coasts of India, affecting the population living along the coasts and infrastructure and inland areas. The extent of the affected inland areas depends on the intensity of the cyclone. On 12 October 2014, a strong cyclone “Hudhud” hit the east coast of India that caused a high degree of devastation along the coast. The impact of this cyclone was seen up to the Himalayan region. Detailed analysis of satellite and ground data show a strong coupling between land-ocean-atmosphere associated with the Hudhud cyclone. The contrast between land and ocean temperature was found to be closely related with the formation of the cyclone in the ocean and its movements towards land. Pronounced changes in the ocean, land, atmospheric, and meteorological parameters with the development of the cyclone and its landfall have been observed. Changes in total column ozone (TCO), relative humidity (RH), and volume mixing ratio of CO (CO VMR), water mixing ratio (H_2_O MMR), surface latent heat flux (SLHF), and aerosol optical properties derived from satellite data show characteristic behavior of the Hudhud cyclone.

## 1. Introduction

Many parts of India suffer with numerous kind of climatic hazards, such as droughts, floods, cyclones, heavy rainfall, earthquakes, landslides, desertification, forest fires, dust storms, and others throughout the year [1,2,3,4,5,6,7,8,9,10]. The rapid economic growth and growing population decline natural resources, increase deforestation and anthropogenic emissions that influence the natural stability of Earth [11,12,13,14,15,16]. As a result, the frequency of natural hazards is on the increase [17,18,19,20]. In recent years, India suffers with intense rains, droughts, landslides, floods, and cyclones, influencing the Indian region including coastal areas [21,22,23,24]. According to the Disaster Management Authority of India, more than 4000 people lost their lives and more than 30 million people were affected by disasters during 1990 to 2000 [25]. In the northern parts of India, the whole region suffers with poor visibility and air quality over the year, due to dust storms during pre-monsoon season (March–June), crop residue burning during April–May and mid-October/November, and anthropogenic activities during the winter season [26,27,28,29,30,31,32]. In the coastal region of India, cyclones have caused severe destruction compared to any other disasters [33,34]. Tropical cyclones originated over warm oceans [35,36,37] impact the weather system. Taking energy with oceans, these complex and intense weather systems may mature into massive vortices with swirling winds, intense clouds, and torrential rains. The movement of cyclones towards land until landfall and afterwards, cause large-scale destruction to life and properties over the coastal and inland regions. In India, the mid and southern parts are surrounded by ocean; the Arabian Sea in the west, the Bay of Bengal in the east, and the Indian Ocean in the south. During the July to September period, low-pressure systems are formed that lead to the formation of severe tropical cyclones. Hoarau et al. [38] observed 21 intense cyclones during 1980–2009, and the decade from 1990 to 1999 was very active in which 11 intense cyclones occurred. The long Indian coastline, about 5700 km, is vulnerable to the landfall of these tropical cyclones. The east and west coasts suffered with frequent tropical cyclones in the past [39,40]. The southwestern part of India suffered with many cyclones in the past and many of them claimed enormous damage to many lives. In 1999, the Odisha cyclone was one of the strongest cyclones in Indian history, killing 10,000 people [39]. Cyclones/typhoons/hurricanes are known influence the ocean productivity, ocean parameters, and also atmospheric properties [20,36,37,39,41,42]. Recently, Pathakoti et al. [43] have observed exchanges between the stratosphere and troposphere causing changes in ozone concentrations. Further, Chow et al. [6] found poor air quality associated with tropical cyclones in China. 

In this paper, detailed analysis of satellite and model data have been carried out to study the influence of the Hudhud cyclone over atmospheric and meteorological parameters, from the day the cyclone was formed in the Bay of Bengal and its landfall at the east coast of India and further movement over land and northwards. Detailed analysis shows pronounced changes in meteorological, aerosol, and atmospheric parameters along the tracks of the cyclone, from south-east to northern parts of India.

### 1.1. Hudhud

**Figure 1:**
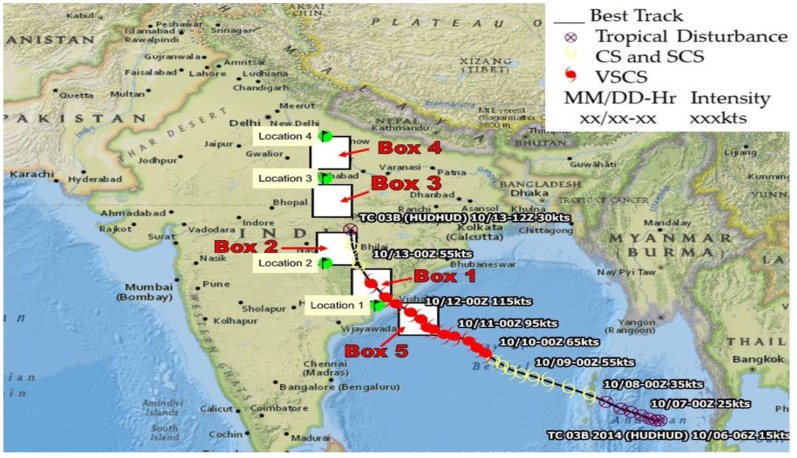
The Hudhud cyclone track during 7–13 October 2014; the cyclone moved northward after landfall, damaging large areas from heavy rainfall. The track is shown from the day of the cyclone formation (7 October 2014) until 13 October 2014 (track of cyclone shown in this figure is taken from Joint Typhoon Warning Centre, (//www.usno.navy.mil/NOOC/nmfc-ph/RSS/jtwc/best_tracks/2014/2014s-KMZs/JTWC_BestTrack_Climatology_2014.kmz). The white squares show the location of five 2^°^ × 2^°^ boxes, as shown in Table 1, and four locations, as shown in Table 2, for the detailed analysis of various parameters. CS: cyclonic storm; SCS: severe cyclonic storm; VSCS: very severe cyclonic storm.

This cyclone was named Hudhud based on the Arabic word which means Hoopoe bird. On 7 October 2014, a low-pressure region was upgraded to a depression near the Andaman Sea, which turned into a cyclonic storm on 8 October 2014 and made its first landfall over Andaman Nicobar in the morning at 08:30, afterwards it moved west-northwest, as shown in Figure 1. It intensified into a severe cyclonic storm (SCS) into the southwest Bay of Bengal in the morning of 9 October and further into a very severe cyclonic storm (VSCS) in the afternoon of 10 October 2014 as shown in Figure 1.

**Figure 2:**
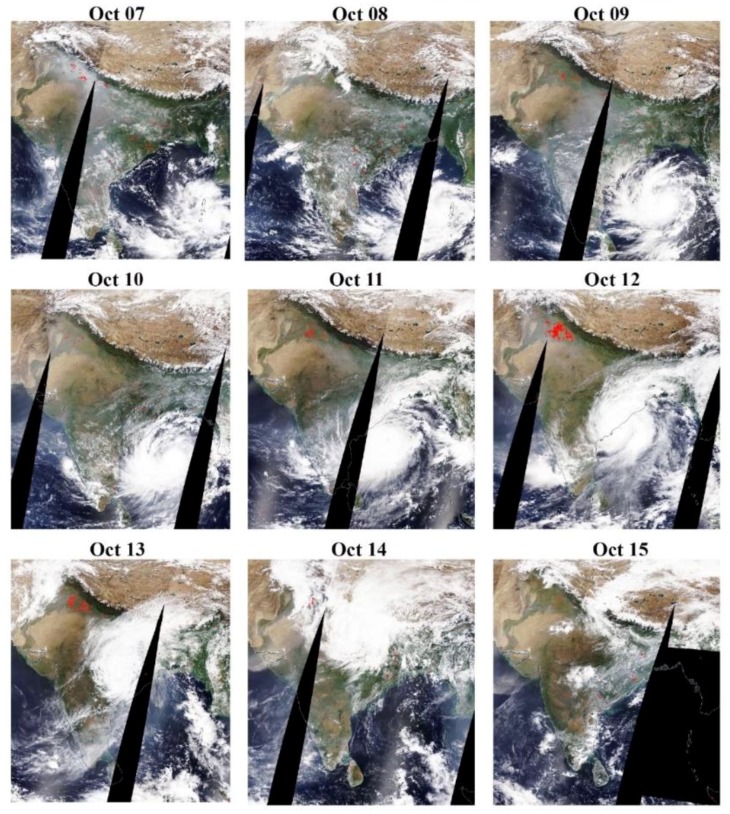
Moderate-resolution imaging spectroradiometer (MODIS) Terra sensor images (7–15 October 2014) showing progress of the Hudhud cyclone and impact over India and Bangladesh. Satellite image (12 October 2014) shows impact of cyclone covering Andhra Pradesh and Odisha states (https://worldview.earthdata.nasa.gov/).

Moving continuously in the northwest direction, the cyclone intensified and reached its maximum intensity in the early morning of 12 October 2014, with the wind speed more than 180 km/h over the west parts of the Bay of Bengal, off the Andhra Pradesh coast. On 12 October 2014, Hudhud crossed the north Andhra Pradesh coast near Visakhapatnam city around 12:00–13:00 with the same intensity. After landfall, the cyclone moved towards northwest with the same speed for some time and gradually the speed weakened. Till the evening on 12 October 2014, the cyclone became a severe cyclonic storm until midnight and afterwards weakened into a cyclonic storm. In the early morning of 13 October 2014, the effect of the cyclone weakened, and changed further into a deep depression by the evening. On 12 October 2014, the eye of cyclone was clearly seen over Visakhapatnam and Odisha, as shown in Figure 2 that persisted on 13 October 2014 causing heavy rainfall in the surrounding areas. The cyclone reached the eastern parts of Uttar Pradesh (northern parts of India), a well-marked low-pressure area on 14 October 2014 causing heavy rainfall in the northern parts covering Uttar Pradesh province and Nepal. Hudhud caused heavy to extreme rainfall along its path that caused large-scale structural damage to the power/water supply and telecommunication infrastructure over Andhra Pradesh and Odisha [44]. As the cyclone progressed after landfall, due to high wind speed, trees were uprooted, disturbing traffic and roads for a few days before the uprooted trees were cleared from the roads. In the state of Andhra Pradesh, over 9 million people living in over 7285 villages in 4 coastal districts were affected and 61 human causalities were reported [44]. The timely warning by the Government of India and the State Government meant people were evacuated from the likely to be affected areas, and as a result, the causalities were minimal. About 112,850 houses were damaged partially or fully, and about 752,540 households were affected severely. It was estimated that 13.25% of agricultural Kharif crops and 31.1% of vegetation was damaged. The total ground biomass of the agricultural crop area was estimated to be 31.57 t/ha and total loss of biomass was about 4.2 t/ha [45].

### 1.2. Study Area

This cyclone severely affected inland after its landfall, heavy rains were recorded from southwest Andhra Pradesh to Nepal and Uttar Pradesh in the north, as shown in Figure 1. We considered five boxes (size 2^°^ × 2^°^, coordinates of boxes are given in Table 1), four boxes on land covering Andhra Pradesh, Chhattisgarh, Madhya Pradesh, and Uttar Pradesh, and Box 5 over the ocean, as shown in Figure 1, close to Visakhapatnam. The track of the cyclone is shown in Figure 1 with a black line and color symbols, the maximum velocity of wind lies at the center of the cyclone, as shown in Figure 1 and Figure 2. We have considered four specific locations; the center longitude and latitude are given in Table 2 and cover an area of 1^°^ × 1^°^ surrounding the four locations.

**Figure 3:**
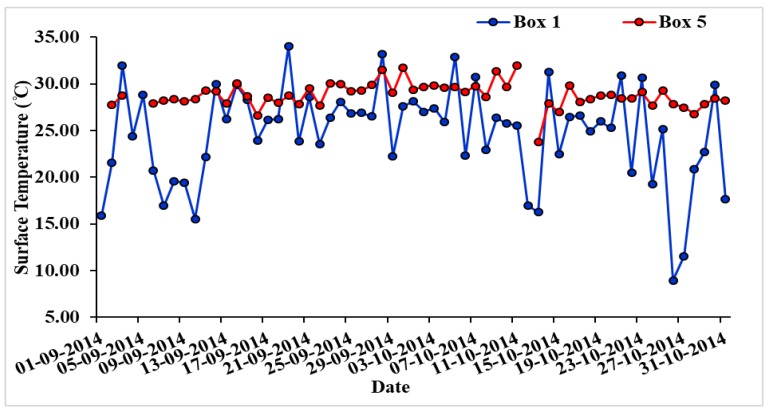
Temporal variation of surface temperature over land (Box 1) and the ocean (Box 5) during 1 September–31 October 2014.

Figure 3 shows the daily mean surface temperature (AIRS data) over land (Box 1) and ocean (Box 5) for the period 1 September–31 October 2014. The average temperature in the month of September 2014 was 25.19 °C on land and 28.90 °C over the ocean, and due to the development of the tropical cyclone, temperature increased to 26 °C over land (Box 1) and 29.89 °C over the ocean (Box 5) during 1–12 October 2014, showing warming of the ocean and land. The land and ocean temperature in the same period in the year 2012/2013 over the same box were about 1.12 °C (land) and 2.24 °C (ocean) lower in the year 2012, and 0.40 °C over land and 1.17 °C over the ocean, lower in the year 2013 compared to the period 1–12 October 2014 during development of the cyclone. A warm ocean is the favorable condition for the formation of cyclones/hurricanes/typhoons [20]. 

The contrast between surface temperatures of ocean and land plays an important role in intensifying tropical cyclones [38,39]. The daily mean surface temperature on land (Box 1—over Vishakhapatnam and nearby regions) and ocean (Box 5—over the sea region near Vishakhapatnam) for the period 1 September–31 October 2014 are shown in Figure 3. Coordinates of all the five boxes are given in Table 1. The average temperature in the month of September 2014 was 25.19 °C on land and 28.9 °C over the ocean, and during 1–12 October 2014, the average temperature increased to 26 °C over land (Box 1) and 29.89 °C over the ocean (Box 5) which clearly shows warming of the ocean and land in the first half of October. 

The motion of a weak pressure wave from east to west develops tropical cyclones under favorable conditions over the Bay of Bengal. Figure 3 shows enhancement of surface temperature of ocean and land with a positive slope during 1–11 October 2014. The higher sea surface temperature with slightly low land temperature in early October provides favorable conditions for an intense cyclone. The deep convective processes involved with cyclones are likely to be associated with the changes in atmospheric and meteorological parameters over the Indian continent, especially along the cyclone track.

## 2. Method and Materials

Hudhud was a very severe cyclonic storm in 2014—it was even one of the deadliest cyclones after the 1999 Odisha super cyclone [36]—and an important factor was that it affected large areas even in the northern parts of India and Nepal from the recorded rainfall. We have analyzed various parameters, which were associated with the formation of the cyclone, and also those which were likely to be affected by the cyclone. The list of parameters and sources are given in Table 3. 

We have used Panoply software for plotting various spatio-temporal parameters for detailed analysis. Panoply software (//www.giss.nasa.gov/tools/panoply/) is developed by NASA based on a Java application that allowed us to make plots from netCDF, HDF, and GRIB datasets. The output of Panoply is in image form which is used in our study. 

### Data Used

The sea surface temperature (SST) anomaly data (resolution 0.25° × 0.25°) has been derived from the Optimum Interpolated SST dataset, obtained from NOAA NCDC (https://www.ncdc.noaa.gov/oisst). This is a gridded data set based on satellites, ships, and marine buoy measurements [46]. Daily mean relative humidity (RH), omega, wind speed and direction, and surface latent heat flux (SLHF) have been derived from NCEP/NCAR reanalysis data. RH and omega data are available with 2.5° × 2.5° spatial resolution whereas SLHF is available with 2° × 2° resolution (https://www.esrl.noaa.gov/psd/data/gridded/data.ncep.reanalysis.html). We have analyzed the vertical profile of relative humidity at four different locations along the path of the cyclone track: location 1–4 shown in Figure 1, Table 2. The choice of coordinates depended on the final data available. We have analyzed the spatial variation of omega to understand the vertical wind direction, SLHF, and vector wind at 700 hPa pressure level. 

Surface temperature, tropopause pressure, total column ozone (TCO), H_2_O Mass Mixing Ratio (H_2_O MMR), and CO volume mixing ratio (COVMR) have been taken from AIRS (Atmospheric Infrared Sounder) satellite Level-3 data. We have considered the average of ascending (daytime) and descending (nighttime) mode data; the ascending mode is when the satellite passes south to north, whereas the descending mode is when the satellite passes from north to south over the Earth’s surface. In ascending mode, the local pass time is around 13:30 (daytime) and in descending mode, the local pass time is around 01:30 (nighttime). Satellite data used in the present paper were downloaded through the NASA Giovanni portal (https://giovanni.sci.gsfc.nasa.gov/giovanni/). Aerosol optical depth (AOD) and Ångström exponent (α) data (Version 6.1) were taken from the moderate-resolution imaging spectroradiometer (MODIS) Terra and Aqua sensors. 

We have also taken the AERONET (Aerosol Robotic Networks) Program (http://aeronet.gsfc.nasa.gov/) data to study aerosol parameters measured from a ground sun photometer and also to validate aerosol optical parameters retrieved from satellites [47]. The sunphotometer deployed under the AERONET is calibrated time to time by the NASA AERONET team. The AERONET data was extensively used to study aerosol characteristics (physical and chemical). We have used AERONET data (Version 3, Level 2) from Kanpur (N-26.51, E-80.23). We have studied total column aerosol properties such as AOD (τ_a500nm_), Ångström exponent (α_470–870nm_), total water column, and single scattering albedo (SSA) over Kanpur using Kanpur AERONET data, during October 2014. The methodology of retrieval of aerosol parameters from AERONET stations have been discussed in detail by Dubovik and King [48] and Dubovik et al. [49].

## 3. Results and Discussion

### 3.1. Spatio-Temporal Variability of SST Anomaly

**Figure 4:**
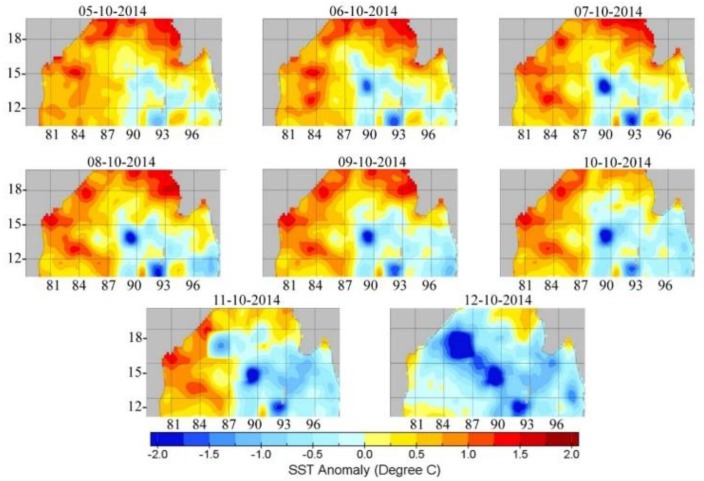
Sea surface temperature anomaly over Bay of Bengal and Andaman Sea. Intense warming is observed near Visakhapatnam coast during 7–11 October 2014.

The formation of the Hudhud cyclone was seen for the first time on 7 October 2014 in the Andaman Sea and landfall occurred on 12 October 2014 near Visakhapatnam, Andhra Pradesh, with a maximum speed of 185 km/h and a minimum pressure of 960 mbar at the center of the cyclone. Clear warming is observed over the ocean from AIRS surface temperatures, as shown in Figure 4. To get more insight into the change in ocean temperature, we have analyzed sea surface temperature anomaly (SST) since 5 October 2014. Strong heating is clearly visible near to the land–ocean boundary during 07–11 October which reflected a higher SST anomaly (more than +1.5 °C) that caused intensification of cyclonic circulation [39], and cooling is left behind by the cyclone. No further heating was seen over the ocean, as further heating of the ocean sometimes causes a second landfall. Due to cooling of the ocean’s surface, the cyclone penetrates deep inside the land as it conserves a continuous east to west circulation of air mass. 

### 3.2. Relative Humidity

**Figure 5:**
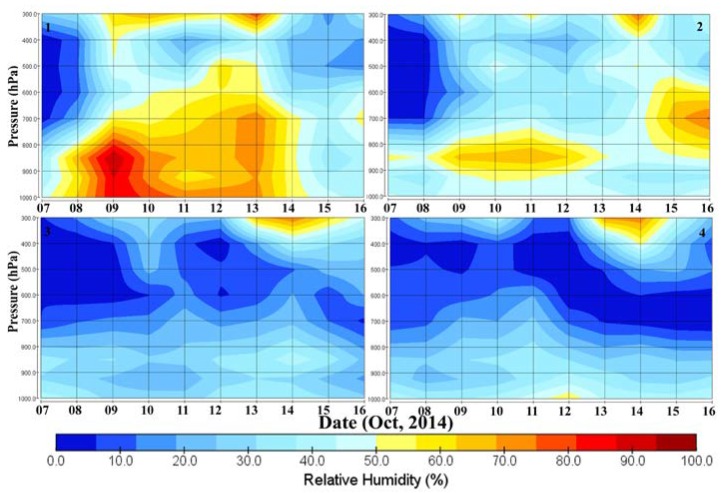
Vertical profile of relative humidity (RH) at four locations (1, 2, 3, and 4) along the track of the cyclone and at the location in the Indo-Gangetic Plain.

Relative humidity plays an important role in circulation of the atmospheric convection cycle during the movement of a tropical cyclone, so we analyzed the vertical profile of relative humidity before and after the cyclone. Figure 5 shows the vertical profile of RH at the different pressure levels of 1000–300 hPa for the period 7–16 October 2014 over four specific locations (locations 1, 2, 3, and 4) along the track of the cyclone and over the location in the Indo-Gangetic Plain (IGP). At the first location (near to Box 1), RH is more than 60% from surface (1000 hPa) to the pressure level 600 hPa during 8–14 August 2014 and from 10% to 40% on 7 October from surface to upper troposphere (UT). On 9 October 2014, the RH is more than 90% at the pressure levels of 850 to 900 hPa; again, the RH was observed to be higher (60%) at UT. The contrast between RH is smaller on 12 October 2014 at the day landfall occurred near the coast of Visakhapatnam. A gradual decrease in vertical profile of RH was observed during 8–12 October and later on RH increases as this is a severe cyclone that caused recorded rain. At the second location (near Box 2), more than 60% RH was observed at the pressure level 850 hPa in the morning of 13 October 2014, and at the higher altitude (low pressure level), RH was observed to be low.

RH was observed to be very high at a narrow zone on 13 October, and on 14 October, high RH (more than 60%) was observed at a higher altitude. Over locations 3 (near to Box 3) and 4 (near to Box 4), low RH (<40%) was observed at a lower altitude and high (70%) relative humidity was found at a higher altitude (at the pressure level 400 to 300 hPa) since late night of 12–15 October—especially on 14 October as RH varied more than 70%. Such changes in RH indicate a strong vertical convection motion of the Hudhud cyclone that persisted until 15 October and also the effect was observed over inland with heavy rainfall.

### 3.3. Omega

**Figure 6:**
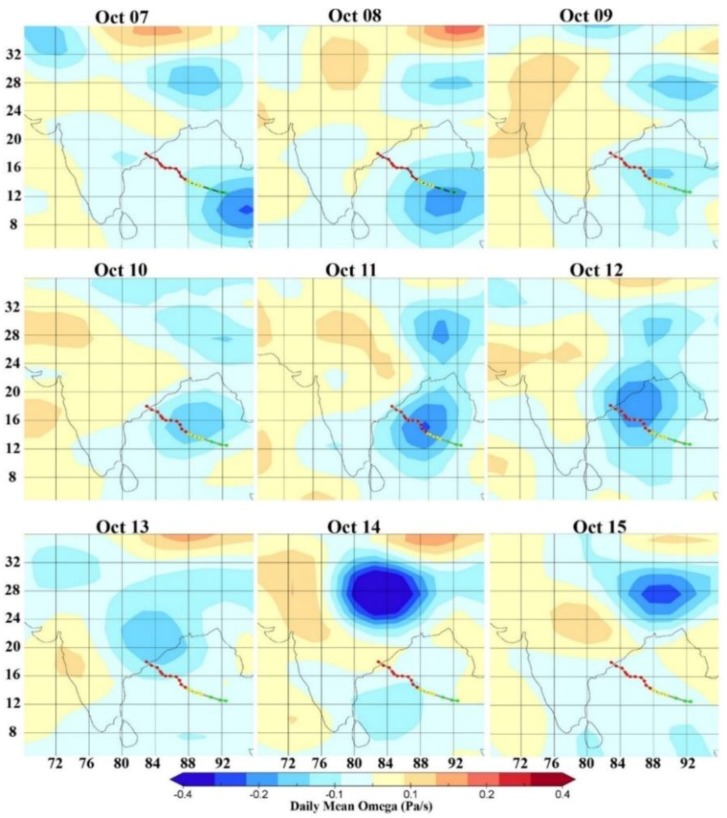
Spatio-temporal variations of omega during 7–15 October 2014 at 700 hPa pressure level along the track of cyclone Hudhud.

Figure 6 shows spatio-temporal variations of omega over India and the Bay of Bengal during 7–15 October 2014 at 700 hPa. On 7 and 8 October, omega showed large negative values when the cyclone started building near Andaman Nicobar. On 10 October, omega values are not negative, showing the fast surface motion of the cyclone towards the coast of Visakhapatnam; afterwards omega showed highly negative values on 11 and 12 October due to the high updraft motion of air-mass over the ocean which led to heavy rainfall over land. On 14 October, over a large area (8^°^ × 8^°^) over Box 4 in the Indo-Gangetic Plains, omega was found to be less than −0.4. Heavy vertical motion with slow forward velocity caused heavy rains over this region over Uttar Pradesh, Bihar, and Nepal during 14 and 15 October 2014 as the cyclone moved northward. The satellite image of the cyclone clearly shows the coverage of the area affected by the movement of cyclonic winds.

### 3.4. Wind Speed and Direction

**Figure 7:**
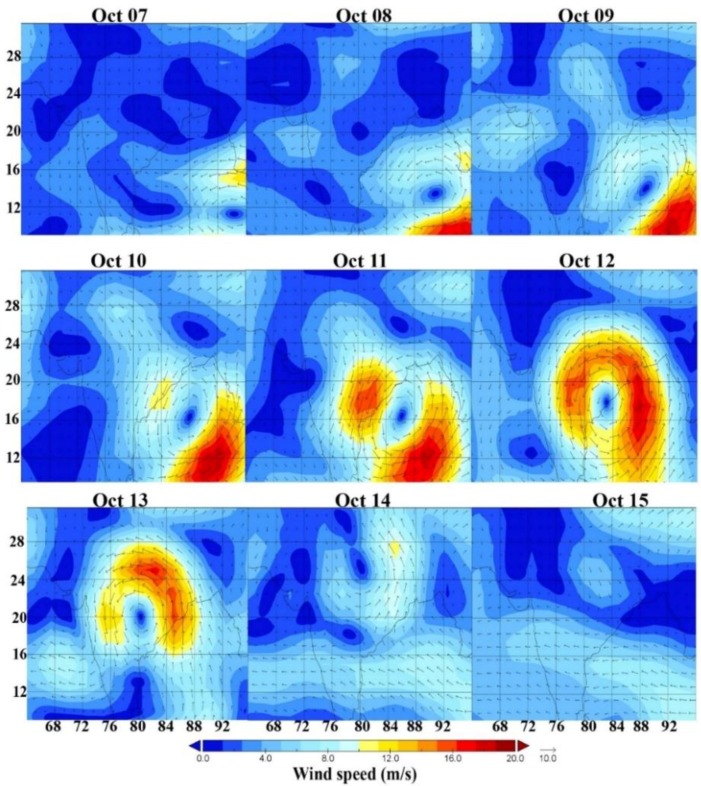
Wind speed and direction derived from NCEP/NCAR reanalysis data at the pressure level 700 hPa over India and the Bay of Bengal.

Figure 7 shows wind speed and direction during 7–15 October 2014; wind circulations show an anticlockwise pattern during 7–14 October 2014. High cyclonic wind was seen around the eye of the cyclone, while low wind velocity was within the eye and its surroundings. Intense high wind affected Andhra Pradesh and Odisha, starting from 11 October. High wind velocity due to the cyclone was observed in the northern parts as of 9 October that affected wide areas in the northern parts of India, covering parts of Uttar Pradesh, Bihar, and west Bengal and Nepal causing heavy rainfall on 14 October.

### 3.5. Surface Latent Heat Flux (SLHF)

**Figure 8:**
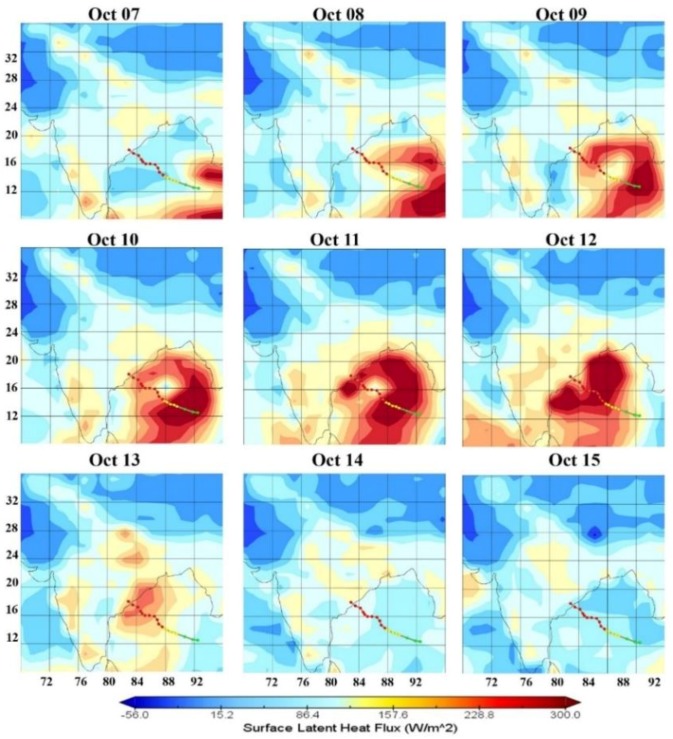
Spatio-temporal variation of surface latent heat lux (SLHF) during 7–15 October 2014. The colored dotted line shows the track of cyclone Hudhud.

Surface latent heat flux (SLHF) depends mainly on sea surface temperature and wind speed at 10 m height. Figure 8 shows the spatial variation of SLHF during 7–15 October 2014: 96.7 W/m^2^ SLHF at the center of cyclone and its surroundings on 7 October 2014 and more than 300 W/m^2^ for favorable conditions for the formation of the cyclone.

The average wind around this region was more than 60 km/h with a sea surface temperature of more than 30 °C. The eye of the cyclone moves forward with a difference in SLHF of more than 200 W/m^2^, which corresponds to the outer shell of the cyclone to convert from a cyclonic storm to a very severe cyclonic storm. On 13 October, a correspondingly high SLHF region was formed near the coast of Visakhapatnam, Madhya Pradesh, and eastern parts of Uttar Pradesh, causing heavy rainfall on 14 October 2014. All the above parameters indicate that the atmospheric conditions were perfectly suited to form a very severe cyclonic storm on 12 October 2014 near to the coast of Vishakhapatnam. This VSCS caused large-scale destruction over coastal parts of India and as the deep depression conditions were formed over central and northern parts of India and Nepal, it caused heavy rainfall. Further, we have discussed the changes in other parameters in association with these cyclonic conditions.

### 3.6. Tropopause Pressure

**Figure 9:**
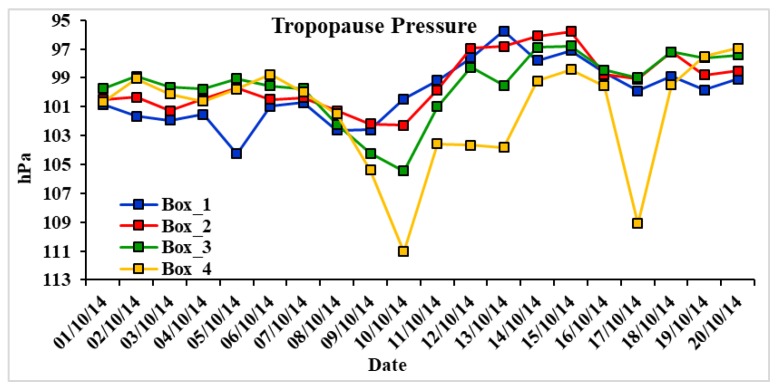
Temporal variation of tropopause pressure over Boxes 1, 2, 3 and 4.

The tropopause height (TH) is affected by radiation and baroclinic eddies and these parameters are highly influenced by cyclones. Figure 9 clearly shows lower tropopause over Box 4 affected by cyclone Hudhud on 10 October. The average tropopause pressure during 1–20 October 2014 over box locations 1, 2, 3, and 4 are 100.06, 99.34, 99.52, and 101.41 hPa, respectively. The height of tropopause shows higher values over all boxes with the approach of the cyclone towards land. On 7 October, tropopause pressure in Box 1 is 102.62 hPa that reduces to 95.76 hPa on 13 October, similarly in Boxes 2 and 3, tropopause pressure reduces to 95.78 hPa and 96.80 hPa on 15 October from 102.27 hPa and 105.44 hPa on 10 October. In Box 4, tropopause height is observed to be lowest (111.06 hPa) on 10 October that recovered to 98.42 hPa due to the cyclonic conditions. On 17 October, again tropopause pressure reduces to 109.11 hPa due to heavy rainfall at a higher altitude in Nepal and in the northern parts of India, followed by a sudden change in atmospheric temperature. The height of tropopause is important as it directly affects the concentration of ozone in the upper troposphere due to vertical cyclonic disturbances. 

### 3.7. Total Column Ozone (TCO)

**Figure 10:**
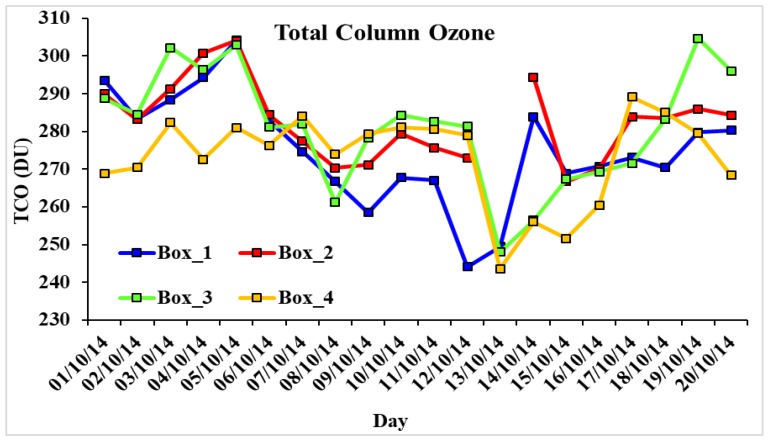
Total column ozone (TCO) over all four 2^o^ × 2^o^ boxes, showing a decrease in TCO on October 12 in Box 1 and October 13 in Boxes 3 and 4. The data is not available in Box 2 for 13 October.

The average total column ozone (TCO) from day and nighttime derived from AIRS data over all four boxes is shown in Figure 10 for the period 1–20 October 2014. As data was not available for each day, the average of day and nighttime are shown to get a more detailed analysis of total column ozone (TCO), which shows a decline in all the four boxes as the cyclone approached land from 6 October; the lowest (244.13 DU) was in Box 1 on 12 October as the cyclone landfalls near to Box 1, close to Visakhapatnam. As the cyclone moves towards the inland, TCO in other regions reaches to the lowest values of 243.60 and 248.00 DU in respective regions (Boxes 3 and 4). TCO values were not available in Box 2 on 13 October 2014. 

TCO recovers on 14 October 2014. During such strong cyclones, pollutants over the lower troposphere (LT) can reach up to the upper troposphere (UT) and in the lower stratosphere affecting the TCO [42]. The change in temperature and relative humidity allow stratospheric ozone to transfer to the troposphere leading to enhancement in tropospheric ozone, which then reacts with chemical species, affecting the air quality [6] and causing a decrease in concentration of TCO. 

### 3.8. Variability of CO VMR

**Figure 11:**
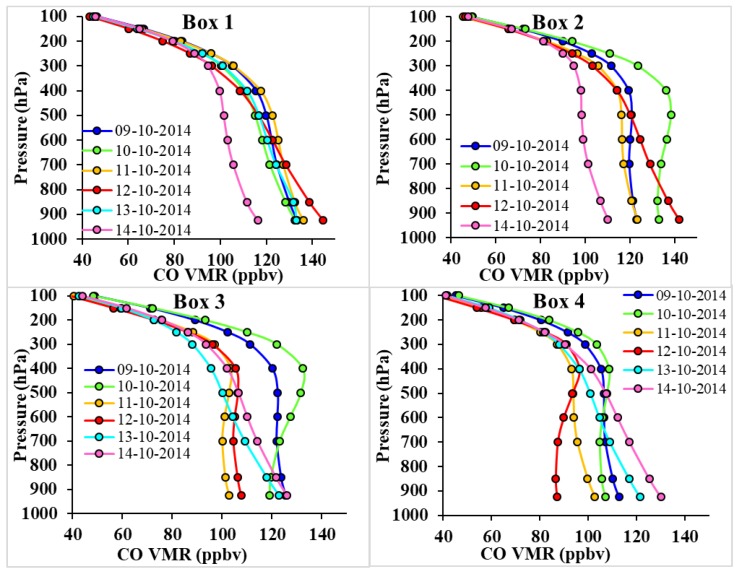
Vertical profiles of CO volume mixing ratio for 9–14 October 2014.

Cyclones are an energetic phenomenon with a strong updraft and downdraft motion that affects the concentration of ozone creating atmospheric imbalance. Due to cyclonic conditions, the concentration of CO volume mixing ratio may increase at higher altitudes. Figure 11 shows the vertical profile of CO volume mixing ratio over all four boxes during 9–14 October 2014. The CO VMR reduced with the height in Box 1 when the cyclone affected the area but in other boxes, the CO VMR was observed to increase up to 400 hPa pressure level prior to and after the cyclone. CO concentration is found to be high due to the cyclone. In Boxes 1 and 2, the CO concentration, respectively, was observed to be highest at 144.70 ppbv and 142.03 ppbv on 12 October, Boxes 3 and 4 show the highest CO VMR, respectively, observed to be 126.10 ppbv and 126.41 ppbv on 14 October 2014, close to the surface (925 hPa). Such high concentrations of CO VMR was likely due to the transport of air mass from surrounding areas with the movement of the cyclone.

The CO VMR data was not available for 13 October 2014 in Box 2. Due to cyclone Hudhud, a higher CO concentration was observed near the surface in comparison to higher altitudes. Changes in the vertical profile of CO VMR over different boxes show the effect of movement of air mass with the cyclonic winds.

### 3.9. Variation of H_2_O MMR

**Figure 12:**
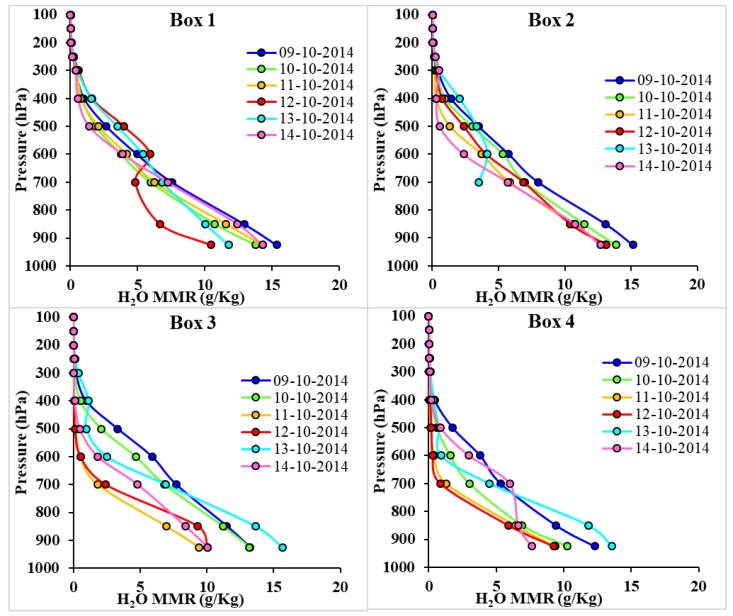
Vertical Profile of H_2_O MMR for 9–14 October 2014.

Strong cyclonic conditions with the approach of the cyclone towards land influence the H_2_O mass mixing ratio (H_2_O MMR); the values were enhanced close to the surface and increase at higher altitudes, as shown in Figure 12. The vertical profile of H_2_O MMR for Boxes 1–4 are shown in Figure 12. In Box 1, the H_2_O MMR is 5.92 g/kg at 600 hPa on 12 October, which is higher (<1.67 g/kg) compared to the other days at this pressure level and lower at the surface (10.45 g/kg at 925 hPa lower than the average value of 1.57 g/kg). The H_2_O MMR is clearly higher near the surface before the cyclone which contains latent heat to help the cyclonic conditions. In Box 2, similar variations are observed on 13 October, where the H_2_O MMR is higher (3.36 g/kg) at 500 hPa corresponding to other days and lower at 700 hPa (3.52 g/kg). Data is not available at lower altitudes (higher pressure levels due to cyclonic disturbance) on 13 October in this region.

In Box 3, enhancement in the H_2_O MMR is observed at a lower altitude (high pressure level) and is highest (15.65 g/kg) at the surface level (925 hPa). Higher water content before the cyclone kept a large amount of latent heat that helped the forward motion of the cyclone, and as the cyclone hit a particular region, the vertical profile indicated towards cyclonic conditions and the water content is found to be higher at higher altitudes in comparison to the surface.

### 3.10. AOD, Angstrom Exponent (AE), and Total Column Water (TCW)

**Figure 13:**
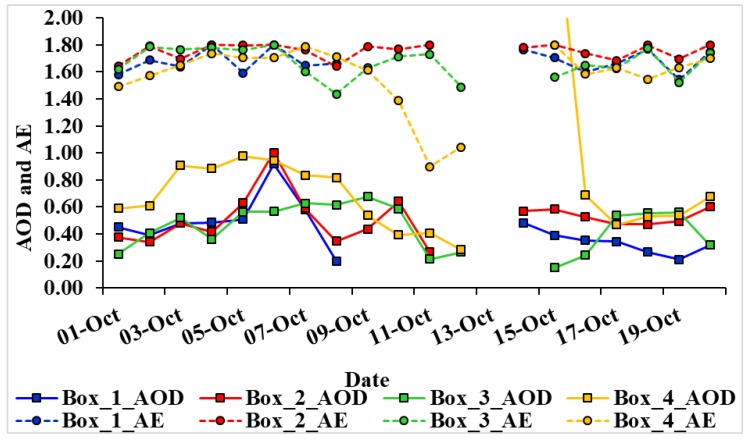
Daily variations of AOD and AE for the period 1–20 October 2014 over Boxes 1–4.

In Figure 13, we have shown temporal variations of AOD and AE for the period 1–20 October 2014 using MODIS Terra and Aqua data. The large variations in aerosol properties was mainly attributed to the wind speed and direction associated with the cyclone bringing more marine air masses over the region during 8–14 October. Due to the cyclonic conditions, daily data was not available at different dates, so we have considered average MODIS Terra and Aqua data.

In Box 1, the average AOD and AE during 1–20 October 2014, respectively, are 0.42 and 1.68; in Box 2, AOD shows 0.51 and AE 1.75; in Box 3, AOD 0.45 and AE 1.67; and in Box 4, AOD shows 0.78 and AE 1.57. The average AOD is highest in Box 4 with higher AE values due to air mass bringing pollutants and also due to crop burning in the northern part of India during October–November [7,24,25,26]. Prior to 6 October, AOD and AE values in Boxes 1–3 show anthropogenic aerosols and in Box 4, the effect of crop residue burning. On 6 October, AOD increases in Boxes 1 and 2, 0.92 and 1.00, and low values in Box 4. A rapid decrease in AOD values with a decrease in AE suggests the mixing of marine air mass due to southeasterly winds associated with the cyclone. In Box 3, AOD values show an increase until 9 October 2014, due to the mixing of a northwesterly air mass. After 9 October 2014, northeasterly winds bring marine air mass and AOD decreases with an increase in AE. In Box 4, a sharp decrease in AE with low AOD values shows a strong mixing of anthropogenic aerosols, biomass aerosols, and dust.

**Figure 14:**
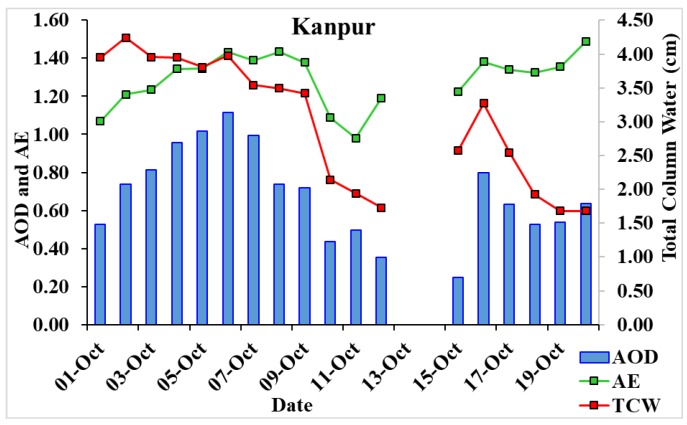
Daily variations of AOD, AE, and TCW at Kanpur, AERONET station during 1–20 October 2014.

In Figure 14, we have shown Kanpur AERONET AOD, AE, and total column water data close to Box 4. AOD in Kanpur shows a higher value (1.12) on 6 October that decreases (0.25) till 15 October with corresponding AE values 1.43 and 1.23. Until 7 October, AOD shows high value (>1) with high AE (>1) value that shows the effect of biomass/crop burning. On 8 October, AOD is less than one with AE (>1), which shows the effect of local anthropogenic emissions. Further, AOD decreases with a decrease in AE until 11 October, showing a mixing of aerosols. We found that total column water decreases before the cyclone hits Kanpur: 1.73 cm on 12 October decreased from 3.98 cm on 6 October, which again shows higher values 2.58 on 15 October and 3.27 on 16 October, due to heavy rainfall on October 14. These results are well in agreement with NCEP/NCAR reanalysis data where RH decreased at the Box 1 location and similar conditions recovered on 15 October that existed prior to the cyclone being formed. We found a sudden increase in AOD, AE, and TCW on 16 October; these values show the effect of the mixing of biomass burning aerosols with suspended water molecules (smog) that started after the rainfall.

### 3.11. Single Scattering Albedo (SSA)

**Figure 15:**
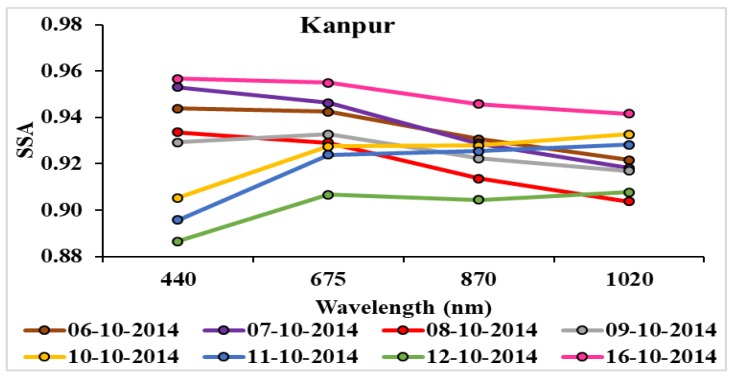
Frequency dependency of single scattering albedo (SSA) at four wavelengths 440, 675, 870, and 1020 at Kanpur AERONET station during 06–16 October 2014. No data is available on 13, 14, and 15 October.

Frequency dependency of SSA, as shown in Figure 15, for the period 6–16 October 2014 shows a decrease in SSA during 6–9 October, with an increase in wavelengths showing dominance of fine particles of local emission. Comparison of SSA on 6 and 7 October shows an increase in SSA found at lower wavelengths and a decrease at higher wavelengths that indicates enhancement in anthropogenic aerosols due to local emission. On 8 October, SSA values further decrease, which indicate enhancement of more absorbing aerosols and this can affect the radiative budget of the atmosphere and help in the intensification of the cyclone [50]. SSA on 9 October indicates a strong mixing of aerosols. On 10 and 11 October, SSA values were found to be lower at lower wavelengths and higher at higher wavelengths that indicate the dominance of dust, and on 12 October, SSA was found to be lowest, indicating the strong mixing of dust with local absorbing aerosols.

On 16 October, SSA was high with an average value 0.95 at all wavelengths, with higher AOD and AE values indicating the presence of suspended water molecules in the atmosphere over Kanpur. Low values of SSA during the period 10–12 October show higher concentrations of black carbon due to mixing of bio mass burning aerosol before the cyclone, and as the cyclone reached near to land, a strong mixing of dust and local emission was clearly seen. 

## 4. Conclusions

Hudhud was one of the worst cyclones that affected the life of many people and caused a huge loss of properties. This cyclone caused a record heavy rainfall not only at the coastal region but also in northern parts of India and Nepal. During October 2014, we found rapid growth in the difference of sea surface temperature and land temperature compared to earlier years. The analysis of several meteorological and atmospheric parameters provided a good understanding of cyclone dynamics and its impacts. A low-pressure region is formed with a change in SST of 1.5 degrees. On 13–14 October, relative humidity was very high at higher altitudes (between 300–400 hPa) over a large region with lower omega (−0.4) which was instrumental in the slow forward and large vertical motion of the cyclone that caused heavy rain. Surface latent heat flux was very high during 7–12 October. All these conditions were favorable conditions for the cyclone that caused heavy damage in coastal parts and also in the central and northern parts of India. As the cyclone reached the coastal region, the height of tropopause increased, which helped stratospheric ozone escape to the troposphere. Pronounced changes were found in TCO during 12–14 October over all four boxes. Due to the increase in tropospheric ozone, the concentration of CO at lower altitudes was enhanced. We found enhancement in the H_2_O mass mixing ratio at the middle troposphere. Aerosol optical properties were also affected as we found the effect of crop burning in coastal regions (Box 1) also mixed with marine aerosols as the cyclone reached the area. Due to the cyclone, we observed strong mixing of anthropogenic aerosols, biomass aerosols, and dust over central and coastal regions. The TCO was found to decrease in the northern parts prior to the cyclone. The results discussed show pronounced changes in ocean, land, atmosphere, and meteorological parameters from the day the cyclone was formed until its landfall and even further. The magnitude of changes in different parameters were clearly observed at different pressure levels on different dates, showing the dynamics of changes along the cyclone track from landfall to northward movement. Such changes clearly show strong coupling between ocean–land–atmosphere associated with the cyclone impact meteorological conditions in widespread areas, affecting weather conditions and bringing excessive rains causing flash floods.

## Figures and Tables

**Table 1:** Coordinates of different boxes.

Box	West	South	East	North
Box 1	81.22	17.69	83.22	19.69
Box 2	79.54	19.84	81.54	21.84
Box 3	79.33	22.67	81.33	24.67
Box 4	79.23	25.51	81.23	27.51
Box 5	83.48	15.69	85.48	17.69

**Table 2:** Coordinates of different locations.

Location	(Center Latitude) North	(Center Longitude) East
Location 1	17.50	82.50
Location 2	20.00	80.00
Location 3	25.00	80.00
Location 4	27.50	80.00

**Table 3:** Parameters used and source of data.

S.No.	Parameters	Source
1	Surface Temperature	AIRS/Giovanni
2	SST Anomaly	NOAA NCDC
3	Relative Humidity	NCEP NCAR
4	Omega	NCEP NCAR
5	Wind Speed and Direction	NCEP NCAR
6	Surface Latent Heat Flux	NCEP NCAR
7	Tropopause Pressure	AIRS/Giovanni
8	Total Column Ozone	AIRS/Giovanni
9	CO VMR	AIRS/Giovanni
10	H_2_O MMR	AIRS/Giovanni
11	Aerosol Optical Depth (AOD)	MODIS and AERONET
12	Angstrom Exponent (AE)	MODIS and AERONET
13	Total Column Water (TCW)	AERONET
14	Rainfall	TRMM/Giovanni

SST: sea surface temperature; VMR: volume mixing ratio; MMR: mass mixing ratio; AIRS: Atmospheric Infrared Sounder; AERONET: Aerosol Robotic Network.
